# Transcriptional and endocrine orchestration of medullary bone formation and mineral turn-over in female chickens

**DOI:** 10.1016/j.psj.2025.105798

**Published:** 2025-09-04

**Authors:** Michael Oster, Hiba Qasir, Henry Reyer, Siriluck Ponsuksili, Nares Trakooljul, Vera Sommerfeld, Markus Rodehutscord, Klaus Wimmers

**Affiliations:** a Research Institute for Farm Animal Biology (FBN), Wilhelm-Stahl-Allee 2, 18196 Dummerstorf, Germany; b Institute of Animal Science, University of Hohenheim, Emil-Wolff-Str. 10, 70599 Stuttgart, Germany; c Faculty of Agricultural and Environmental Sciences, University Rostock, Justus-von-Liebig-Weg 6, 18059 Rostock, Germany

**Keywords:** Calcium turn-over, Maturation, Mineral deposition, Laying period, RNA-sequencing

## Abstract

Medullary bone is deposited in the cavities of avian long bones and serves as a calcium reservoir for successful eggshell mineralization in sexually mature female chicken. Osteoblasts and osteoclasts located in medullary bone respond to endocrine changes, including high estrogen levels at sexual maturation, and represent important targets for improving mineral turn-over in laying hens. In this study, weekly samples of blood and femur to extract medullary bone material were taken from Lohmann Brown (**LB**, *n* = 54) and Lohmann Selected Leghorn (**LSL**, *n* = 54) hens from pullet stage (week 16 of age) until onset of oviposition (week 24). Blood beta **CTX-1** (C-terminal telopeptides of type I collagen) levels increased in week 17 (LSL) and week 18 (LB) until week 21, indicating organic matrix breakdown for initial bone remodelling. Estradiol increased not before week 20 (LSL) and week 21 (LB). Subsequently, medullary bone calcium content increased in week 23 (LB, LSL), whereas the phosphorus content increased in week 22 (LSL) and week 23 (LB). The longitudinal gene expression patterns of medullary bone material across the maturation period showed pronounced synergistic activities to ensure vascularisation, energy metabolism, and ossification. These analyses identified key genes and pathways involved in successful medullary bone formation in both LB and LSL strains, offering potential targets for genetic or nutritional interventions aimed at maintaining efficient mineral turn-over throughout the laying period. Taken together, the cascade-like sequence of bone remodelling, which controls the differentiation and activity of osteoblasts and osteoclasts and ultimately drives the transition from haematopoietic bone marrow in pullets to medullary bone in mature female birds, is initiated earlier in LSL than in LB hens.

## Introduction

Egg production in laying hens is a physiological process that demands a substantial supply of minerals, particularly calcium (**Ca**) and phosphorus (**P**), to ensure animal health, fertility, and effective eggshell formation. Unlike other mineralized compartments such as bone and mammalian teeth, which consist of a cell-organized matrix, eggshells mainly comprise Ca carbonate (**CaCO_3_**) and are based on an extracellular mineralization process. The eggshell formation occurs in the uterus of the hen and is among the fastest mineralization processes known (Nys and Roy, 2018; Rodríguez-Navarro et al., 2015). In high-yielding laying hens, about 2 to 3 g of Ca (approximately 10 % of the total Ca stores of the body) is transported daily to the oviducts for eggshell formation (Nys and Guyot, 2011; Nys and Roy, 2018). In order to cover the high Ca requirement, sufficient Ca must be supplied with the feed, whereby 30-40 % of the required Ca is mobilised from body Ca stores, i.e. medullary bone (Comar and Driggers, 1949; Nys and Guyot, 2011). The medullary part of the bone is a specialized estrogen-induced bone type that is found as a dynamic Ca reservoir mainly in long bones such as femur and tibia of poultry species (Dacke et al., 1993; Kerschnitzki et al., 2014; Whitehead, 2004). Medullary bone may contribute to the fracture resistance of the surrounding cortical bone, but it was not shown to be load-bearing (Fleming et al., 1998). In fact, medullary bone is subject to rapid cycles of resorption and deposition in matured laying hens, ensuring the supply of Ca for successful eggshell mineralization throughout the entire reproductive period (Kim et al., 2012; Van de Velde et al., 1985).

At onset of the laying phase, mineral metabolism necessitates a continuous formation of medullary bone (Dacke et al., 1993; Whitehead, 2004). The critical transition period from pre-laying (week 16) to onset of laying (week 24), in which the hens’ physiology changes from the growth phase to reproductive maturity, is characterized by time-dependent increase of plasma Ca (Sommerfeld et al., 2024) and increased levels of hormones, including estradiol and calcitriol (Omotoso et al., 2021). As poultry approach sexual maturity, estrogen, primarily derived from the growing ovarian follicles, is crucial in initiating medullary bone formation through estrogen receptors (Turner et al., 1993). Consequently, estradiol mediates the stimulation of osteoblast activity (Turner et al., 1993) to deposit a collagenous and non-collagenous protein and lipid matrix, which later mineralizes to form medullary bone. As is known from observations in mammals, estradiol also influences the differentiation and activity of osteoclasts (Kameda et al., 1997; Nakamura et al., 2007), which are significantly involved in bone remodelling through the breakdown of collagens (Blair et al., 1993; Sires et al., 1995). Levels of carboxy-terminal collagen crosslinks (**CTX-1**) are proportional to osteoclastic activity and serve as an indicator of bone turn-over (Rosen et al., 2000).

Previous studies have shown the distinct adaptations of several body compartments for efficient mineral utilization from growing hens to the onset of the laying period. These comprised an increase in Ca intake and utilization (Sommerfeld et al., 2020), increased expression of genes encoding mineral transport in the jejunum (Omotoso et al., 2021), altered miRNA profiles associated with phytate degradation (Ponsuksili et al., 2021; Sommerfeld et al., 2020), and shifts in immune capacity (Schmucker et al., 2021). A number of these phenotypic traits showed a pronounced strain effect between Lohmann Brown (**LB**) vs. Lohmann Selected Leghorn (**LSL**). However, detailed responses to shifts in endocrine environment during the critical phase of sexual maturation on osteoblasts and osteoclasts hosted in the medullary bone are not yet clear and represent promising targets for improving mineral flow in laying hens.

We hypothesize that the dynamics of mineral mobilization from medullary bone in laying hens differ over the sexual maturation period (pullet to layer transition), subjected to the critical shifts in gene expression and endocrine parameters. Therefore, this study investigated weekly changes in gene expression within the medullary bone of the femur in LB and LSL laying hens from pre-laying (week 16 of age) to onset of laying (week 24). In addition, the study analysed the effects of strains and weeks of life on plasma metabolites relevant to mineral utilization and bone remodelling.

## Material and methods

### Ethics and consent to participate

This study was conducted as part of the interdisciplinary Research Unit P-Fowl: Inositol phosphates and myo-inositol in the domestic fowl: Exploring the interface of genetics, physiology, microbiome, and nutrition (https://p-fowl.uni-hohenheim.de/). Newly hatched female chickens were provided by a breeding company (Lohmann Breeders GmbH, Cuxhaven, Germany), with the owners' consent for their use in the research. The animal trial was performed at the Agricultural Experimental Station of the University of Hohenheim, Germany. The experimental protocol was in strict compliance with the German Animal Welfare Legislation and approved by regional Ethics committee, i.e., the Regierungspräsidium Tubingen, Germany (Project no. HOH67-21TE).

### Experimental setup and laying hens

**Fig. 1:**
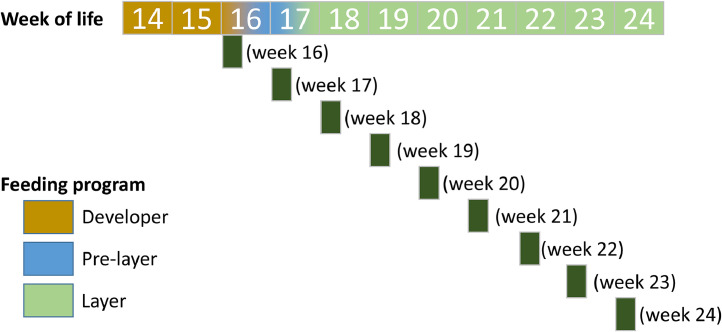
Experimental timeline illustrating the feeding and sampling schedule of LB and LSL hens. The transitions between developer (week 14-16), pre-layer (week 16-17), and layer feed phases (week 17-24) are shown along the timeline. For each sampled strain from week 16 to week 24 (*n* = 10 per strain per week), sampling time points are indicated in dark green.

Both LB (*n* = 90) and LSL (*n* = 90) hens were studied in 9 weekly cohorts of 10 individual birds from week 16 to week 24. Hens were supplied with experimental maize-soybean meal-based diets having all required nutrients in recommended doses, lacking phytase supplementation (Supplementary Table 1). The hens’ feed supply according to sampling regime is given in Fig. 1. Hens were assigned to different feeding programs according to their developmental stage, transitioning sequentially from developer feed (week 14-16) to pre-layer feed (week 16-17) and finally to layer feed (week 17-24). The total Ca concentration was 10.3 g/kg dry matter (**DM**) for developer feed, 22 g/kg DM for pre-layer feed and 35.7 g/kg DM for layer feed. The total P content was 4.9 g/kg DM for developer feed, 5.0 g/kg DM for pre-layer feed and 4.9 g/kg DM for layer feed. The total number of intact eggs laid by each hen up to the time of tissue sampling was recorded.

Hens were euthanized between 09:00 h and 15:00 h through individual stunning with a gas mixture (35 % CO_2_, 35 % N_2_, and 30 % O_2_), followed by decapitation. Trunk blood was collected in lithium-heparin tubes, centrifuged, and the resulting plasma samples were stored at −80°C until analysis. Femur bones were sampled approximately 5 minutes post-slaughtering. Femurs of the left leg were cut at the metaphysis to expose the medullary cavity. The medullary bone including bone marrow was gently separated from the surrounding cortical and trabecular bone with a spatula to minimize contamination and frozen at −80°C until analysis. Femurs from the right leg were kept intact and stored at −20°C until analysis.

### Bone traits

Following thawing overnight at 4°C, femur samples were weighed (*n* = 180). The fracture load of the bone specimens was determined by a three-point bending test using a flexural testing apparatus (WINOPAL Forschungsbedarf, Elze, Germany). In brief, each femur was positioned horizontally with the proximal and distal epiphyses resting on two supports, while the middle diaphyseal region (50 % of the total length) served as the loading point. A vertically aligned loading anvil (50 kg capacity) compressed the diaphyseal region at a constant rate of 2 mm/s until structural failure occurred. Throughout the procedure, a digital monitoring system captured real-time load-displacement data, allowing accurate quantification of fracture strength by analysing the characteristic curve. As the medullary cavity was exposed after the breaking test, medullary bone including bone marrow was carefully separated from cortical and trabecular bone with a spatula to minimize contamination. The medullary bone material was collected in sampling tubes. The remaining femur samples were dried for 48 h at 103°C in a convection oven (VL115, VWR International GmbH, Darmstadt, Germany), cooled in a desiccator and weighed. Ash content was determined at 600°C in a muffle furnace (L40/11/B170, Nabertherm GmbH, Lilienthal, Germany) for 24 h. Ashed femur samples were weighed and pulverized. Following digestion with nitric acid, ground femora were analysed for Ca and P using inductively-coupled plasma optical emission spectrometry as described previously with minor modifications (Shastak et al., 2012). Medullary bone samples were freeze-dried and, after wet microwave digestion with nitric acid, analysed for Ca and P using inductively-coupled plasma optical emission spectrometry. Neither cortical bone nor medullary bone material were subjected to any de-fatting procedures (Shastak et al., 2012).

### **Blood** m**etabolites**

The plasma samples (*n* = 180) were analysed for inorganic P and total Ca using the Fuji DriChem 4000i commercial assays (FUJIFILM Cooperation, Minato, Tokyo, Japan). Hormones were quantified in duplicate using commercially available enzyme-linked immunosorbent assays (ELISA) following the manufacturer’s guidelines. The measured hormones were estradiol (EIA-2693, DRG, Marburg, Germany) and beta CTX-1 (AC-02F1, IDS Immunodiagnostic Systems GmbH, Frankfurt am Main, Germany). Raw data were processed using MARS data analysis software (BMG Labtech, Ortenberg, Germany) with 4-parameter logistic curve analysis.

### RNA extraction and sequencing

About 60 mg of each medullary bone sample (*n* = 108; 6 replicates per strain and week with the same paternal lineage, i.e. 6 roosters) were disrupted using 0.5 mm beads and TRI reagent (Sigma-Aldrich, Taufkirchen, Germany) in a Precellys tissue homogenizer (Peqlab, Erlangen, Germany). The RNA was isolated using the phenol-chloroform-based RNA extraction method. To eliminate DNA contamination, the extracted RNA was treated with DNase I (Roche Diagnostics, Mannheim, Germany) and further purified using the NucleoSpin RNA extraction kit (Macherey-Nagel, Düren, Germany). The RNA concentration was measured using the NanoDrop ND-2000 spectrophotometer (Peqlab), while RNA quality was assessed with the Bioanalyzer 2100 system (Agilent Technologies, Waldbronn, Germany). The individual RNA integrity numbers (RIN) ranged from 6.5 to 8.5, with a mean value of 7.6. The absence of genomic DNA contamination was verified through polymerase chain reaction (PCR) targeting a chicken GAPDH fragment. The RNA libraries were generated using the Illumina Stranded mRNA preparation kit (Illumina, San Diego, CA, USA) and sequenced on the Illumina NextSeq 2000 platform in a paired-end configuration.

### Processing of sequencing data

Quality control and pre-processing of the raw sequencing reads were conducted using FastQC (v0.11.7) and Trim Galore (v0.5.0; https://www.bioinformatics.babraham.ac.uk/projects/). Low-quality reads (mean Q-score < 20) and shorter length reads (< 30 base pairs) were excluded. The remaining reads were aligned to the chicken genome assembly (GRCg6a, Ensembl release 109) using Hisat2 (v2.1.0; http://daehwankimlab.github.io/hisat2/). HTseq (v0.11.2) was used to summarize read counts in order to determine the expression levels of each gene. Data are available from the EMBL-EBI database (www.ebi.ac.uk/arrayexpress) under the accession numbers E-MTAB-15166 and E-MTAB-15167. Each of the femur medullary bone samples yielded an average of 28.36 million paired-end reads for downstream analysis. The arrayQualityMetrics package (R, v3.54) was used to identify potential sample outlier. All medullary bone samples from week 19 of both strains (*n* = 12) were excluded from the analyses due to their distinctly different profiles from the other bone specimen.

### Statistical analysis

For analyses of bone and blood traits, statistical evaluation was conducted using linear models, including week of life within strain as fixed effect (R, version 4.3.0, stats package v3.1-160). Differences between experimental subgroups were evaluated with the aov and TukeyHSD functions, with statistical significance determined at *P*-value < 0.05 (R, version 4.3.0, stats package). Graphical presentation of the results was performed using the ggplot2 package (v3.3.6) in R.

Via the DESeq2 package in R (v1.38.3), gene expression analysis was performed per consecutive weeks within strain and between the same weeks of life across the two strains. Initial filtration comprised the inclusion of genes with 10 or more counts in at least 5 samples. The statistical model comprised strain and week of life as fixed effects. Comparisons focused on (i) strains per week as well as (ii) consecutive weeks per strain. Differentially expressed genes (**DEG**) at *P*-value < 0.01 were reported.

To analyse temporal gene expression patterns across the transition from immature pullet stage to the onset of egg laying in the LB and LSL strains, the **STEM** (short time-series expression miner) Java application was utilized for clustering, visualization, and comparison of gene expression trends (Ernst and Bar-Joseph, 2006). Prior to analysis, raw count data were normalized using variance stabilizing transformation (**VST**) in DESeq2 (R, v1.38.3) to stabilize variance across expression levels. The processed data were then separated by strain, and the median expression level for each gene was computed per week of life. These values were subsequently submitted for STEM analysis, where gene expression profiles were clustered based on similar temporal patterns. Profiles with a statistically significant number of assigned genes were reported. Based on the clustering results, biologically relevant profiles were selected for further interpretation and downstream analysis. The chicken Ensembl-IDs were converted to gene symbols using the online tool g:profiler (https://biit.cs.ut.ee/gprofiler/gost). For data enrichment analysis, genes assigned to STEM profiles 1 to 4 were selected and analysed through g:profiler. Biological Processes of the Gene Ontology (**GO:BP**) were used considering maximal term sizes of 500. Terms with a Benjamini-Hochberg adjusted *P*-value < 0.05 were considered significant. A set of genes assigned to STEM profile 1 and related to ‘ossification processes’ in both LB and LSL strains was selected (GO:0001503) to visualize the relative temporal trends and deviations from the immature pullet stage. The VST-values from week 16 of life were subtracted from each subsequent time point per strain. The numerical differences in expression were plotted as a heatmap (GraphPad Software, San Diego, CA, USA).

## Results

### Bone traits

**Fig. 2:**
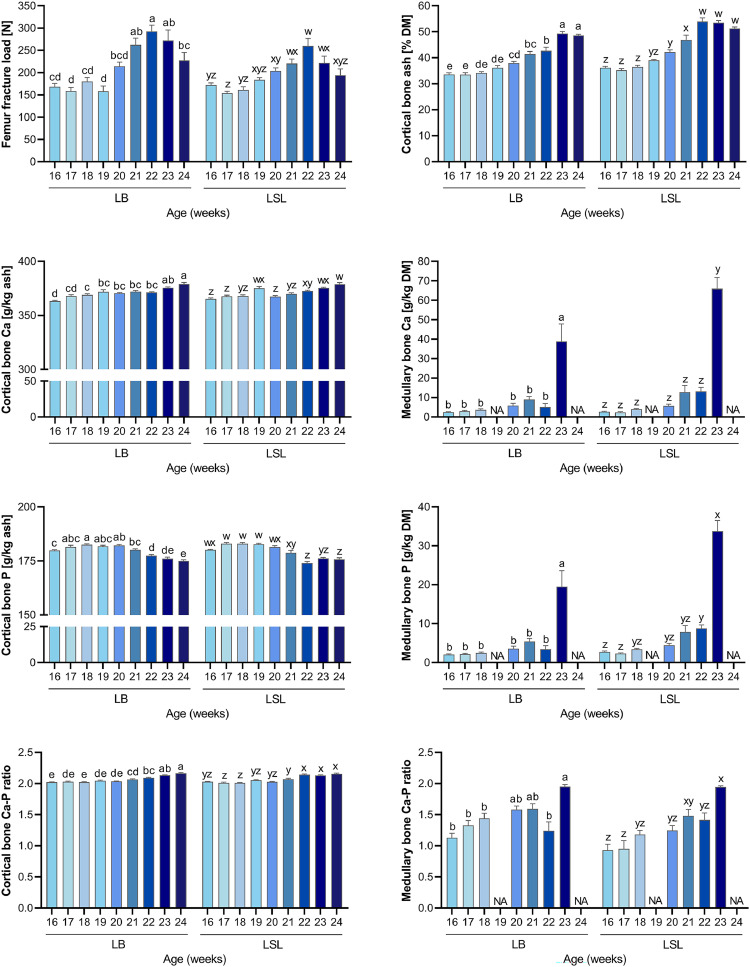
Evaluation of breaking strength, relative bone ash content, and Ca and P contents in cortical and medullary regions of femurs from LB and LSL hen strains throughout the experimental period. Values are presented as mean ± SEM. Different superscripts indicate statistical differences between the weeks within each strain at *P*-value < 0.05 (^a-e^ for LB; ^w-z^ for LSL).

Fig. 2 shows results of functional and structural properties of bone specimen in LB and LSL hen strains from pullet stage (week 16) throughout the onset of laying (week 24). The femur fracture load showed increased levels at week 22 compared to pullet stage in both LB and LSL (*P* < 0.05), which was followed by a decline at week 24 (*P* < 0.05). The relative ash content referring to cortical bone specimen increased with maturation (week 20) and showed a plateau (*P* < 0.05) in week 22 in LSL and week 23 in LB. In cortical bone, the Ca content increased with the lowest levels at week 16 and the highest levels at week 24 in both strains (*P* < 0.05), whereas the P content declined from week 21 onwards in both strains (*P* < 0.05). This resulted in an increasing Ca-P ratio in cortical bone in week 21 (LB, LSL), which further increased in week 22 (LSL) and week 23 (LB), i.e., Ca storage accelerated compared to P storage in cortical bone (*P* < 0.05). The analysis of Ca and P contents in medullary bone data revealed 5 outliers that were deleted from further analysis. In medullary bone, the Ca content remained low until week 22 and increased at week 23 in both strains (*P* < 0.05). Its P content was elevated in week 23 in LB and in weeks 22/23 in LSL hens compared to week 16 (*P* < 0.05). The resulting Ca-P ratio in medullary bone of LB hens increased in week 23 compared to initial values (*P* < 0.05). The LSL hens showed an increased Ca-P ratio in weeks 21 and 23, indicating disproportionately higher Ca deposition than P deposition (*P* < 0.05).

### Blood metabolites

**Fig. 3:**
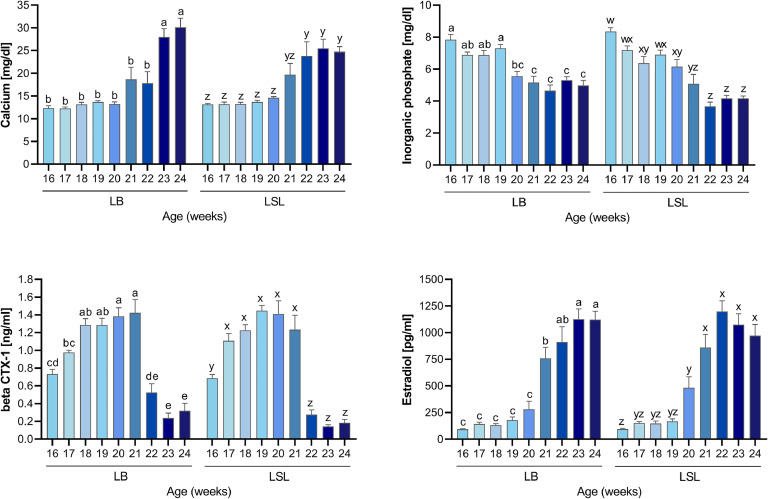
Plasma parameters related to mineral homeostasis in LB and LSL hen strains from pre-laying (week 16) to onset of laying (week 24) phases. Values are presented as mean ± SEM. Different superscripts indicate statistical differences between the weeks within each strain at *P*-value < 0.05 (^a-e^ for LB; ^w-z^ for LSL).

Fig. 3 shows plasma traits related to mineral homeostasis in LB and LSL hen strains from pullet stage (week 16) throughout onset of laying (week 24). A time effect was observed in both strains on all four plasma parameters (*P* < 0.05). Plasma total Ca levels remained stable from weeks 16-22 in LB and from weeks 16-21 in LSL groups, followed by an increase from week 23-24 in LB and from week 22-24 in LSL hens (*P* < 0.05). Plasma inorganic phosphate concentrations dropped from week 20 onwards compared to pre-layer plasma levels in both strains and dropped further at week 22-24 in LSL hens (*P* < 0.05). Plasma beta CTX-1 levels were elevated at week 18-21 in LB and at week 17-21 in LSL hens compared to week 16 (*P* < 0.05). Both strains showed declining beta CTX-1 levels from week 22 onwards. Plasma beta CTX-1 levels were lower at week 23-24 in LB and at week 22-24 in LSL hens compared to initial plasma level at week 16 (*P* < 0.05). Plasma estradiol levels increased at week 21 in LB and at week 20 in LSL hens compared to initial levels at week 16 (*P* < 0.05). The estradiol levels further increased at week 23-24 in LB and at week 21-24 in LSL hens (*P* < 0.05). Notably, egg-laying activity was not observed before week 21. Regarding birds sampled at week 21, the number of hens which started egg-laying activity were 1 out of 10 LSL hens and 0 out of 10 LB hens (week 22: 2 out of 10 LSL hens and 2 out of 10 LB hens). Moreover, 8 out of 10 LB hens have laid eggs in week 23 and 24, respectively, while 9 out of 10 and 10 out of 10 LSL hens have laid eggs in week 23 and 24, respectively.

### Differential gene expression analysis per time point

**Fig. 4:**
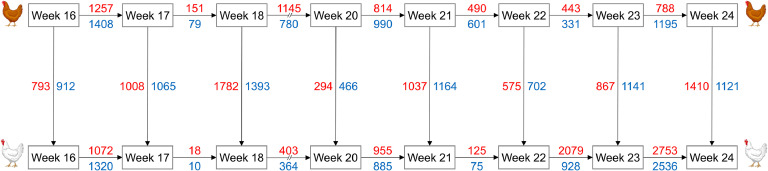
Numbers of differentially expressed genes (DEG) in the femur medullary bone between weeks of age during the transition from immature pullet stage to the onset of lay within LB and LSL strains (horizontal), and between the two strains in each week (vertical). Number of upregulated genes are given in red colour (e.g., 1257 genes with significantly increased abundance from week 16 to week 17 in LB hens), while number of downregulated DEG are given in blue colour (e.g., 1408 genes with significantly decreased abundance from week 16 to week 17 in LB hens).

The differential gene expression analysis across weeks within and between LB and LSL strains revealed distinct transcriptional dynamics (Fig. 4). Comparisons between (i) the two strains at each week (Supplemental Table 2) and (ii) among consecutive weeks within each strain showed pronounced numbers of upregulated and downregulated DEG (Supplemental Table 3, Supplemental Table 4). The strain comparisons per week showed the highest differences in terms of number of DEG at week 18, where 1782 genes were upregulated and 1393 genes were downregulated in LB compared to LSL laying hens. The comparisons between consecutive weeks within strain showed pronounced differences between weeks 16 to 17 in LB and between weeks 23 to 24 in LSL.

### Longitudinal gene expression profiles

**Fig. 5:**
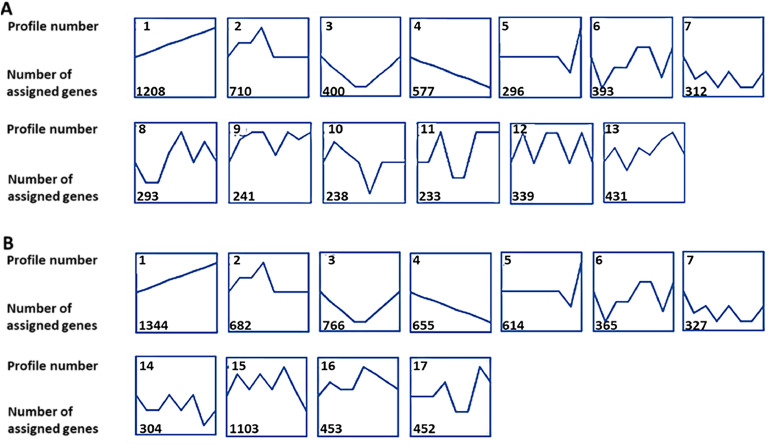
Time series gene expression patterns in the femur medullary bone from week 16 to week 24 in LB (A; *n* = 48) and LSL (B; *n* = 48) laying hens. Each box represents an expression profile with a statistically significant number of assigned genes identified through STEM analysis. The identifying number of each profile is displayed in the top-left corner. The number of assigned genes per profile is displayed in the bottom-left corner. Selected profiles (no. 1, 2, 3, and 4) were chosen for downstream analysis due to their biological relevance. Data referring to week 19 were not included.

Fig. 5 shows the results of the longitudinal analysis of gene expression patterns in the femur medullary bone of LB and LSL laying hens (Supplemental Table 5, Supplemental Table 6). The analyses revealed distinct temporal patterns for different clusters of genes covering the period from week 16 to week 24. LB and LSL strains showed overlapping gene expression trends over time, as displayed in profiles 1 to 7, whereas profiles 8 to 13 were unique for LB and profiles 14 to 17 were unique for LSL hens. Among the observed patterns, profiles 1-4 were selected due to their characteristic expression trends, which either showed continuous changes with age (increasing or decreasing) or distinct shifts during the transition to laying. Specifically, profile 1 showed a steady upward trend from pre-laying at week 16 to the onset of laying at week 24 (LB: 1208 genes; LSL: 1344 genes). Profile 2 exhibited a fluctuating expression pattern, where transcript abundance initially increases until week 20, decreases until week 22, and then plateaus (LB: 710 genes; LSL: 682 genes). Profile 3 initially experiences a pronounced dip until week 20 and starts increasing from week 21 onwards (LB: 400 genes; LSL: 766 genes). Profile 4 follows a declining trend throughout the observed maturation period until onset of laying (LB: 577 genes; LSL: 655 genes).

### Functional enrichment analysis

Table 1 shows GO:BP enrichment analysis of genes with respect to strain in the selected profiles 1-4. The associated gene lists are listed in Supplementary Table 7. In profile 1, both LB and LSL showed significant enrichment in processes such as ossification, collagen metabolic process, skeletal system development, and osteoclast differentiation. The LSL data also included extracellular matrix organization and connective tissue development. According to profile 2, both strains showed similar expression patterns of genes involved in histone modification. Additionally, pathways like regulation of protein-containing complex assembly and blood vessel remodelling were prominent in LB, whereas LSL revealed significant enrichment in DNA replication. Profile 3 showed an association with carbohydrate metabolic process in both laying hen strains. Results showed significant involvement in purine-containing compound transmembrane transport in LB and negative regulation of autophagy and carbohydrate metabolic process in LSL hens. For profile 4, enriched genes of LB hens were primarily associated with pathways involved in oxidative stress and protein regulation, such as reactive oxygen species metabolic process and hydrogen peroxide metabolic process. In contrast, enriched genes in profile 4 of LSL hens were predominantly associated with immune response pathways, such as inflammatory response and regulation of cytokine production.

**Table 1:** Gene ontology biological processes (GO:BP) based on genes assigned to the longitudinal expression analyses per strain (LB, LSL) in profiles 1,2,3, and 4.

Profile	Strain	GO:BP	Term ID	Gene count (term size)	adj. *P*-value
1	LB	Ossification	GO:0001503	46 (241)	1.95E-09
		Collagen metabolic process	GO:0032963	17 (38)	1.81E-08
		Skeletal system development	GO:0001501	55 (346)	2.61E-08
		Osteoclast differentiation	GO:0030316	20 (62)	3.42E-07
		Bone development	GO:0060348	31 (155)	3.04E-06
		Osteoblast differentiation	GO:0001649	28 (138)	1.33E-05
		Cellular response to growth factor stimulus	GO:0071363	54 (406)	3.03E-05
		Response to growth factor	GO:0070848	54 (414)	5.95E-05
		Biomineralization	GO:0110148	22 (97)	6.58E-05
		Plasma membrane bounded cell projection morphogenesis	GO:0120039	48 (353)	9.67E-05
	LSL	Osteoclast differentiation	GO:0030316	20 (62)	2.12E-06
		Ossification	GO:0001503	43 (241)	2.34E-06
		Skeletal system development	GO:0001501	54 (346)	2.92E-06
		Collagen metabolic process	GO:0032963	15 (38)	1.12E-05
		External encapsulating structure organization	GO:0045229	34 (188)	9.33E-05
		Extracellular matrix organization	GO:0030198	34 (188)	9.33E-05
		Extracellular structure organization	GO:0043062	34 (190)	1.22E-04
		Sterol biosynthetic process	GO:0016126	11 (25)	3.40E-04
		Connective tissue development	GO:0061448	31 (174)	5.21E-04
		Myeloid leukocyte differentiation	GO:0002573	26 (132)	6.47E-04
2	LB	Regulation of protein-containing complex assembly	GO:0043254	25 (227)	1.57E-02
		Histone modification	GO:0016570	30 (307)	2.28E-02
		Blood vessel remodeling	GO:0001974	7 (22)	3.61E-02
		Positive regulation of canonical Wnt signaling pathway	GO:0090263	12 (69)	4.26E-02
		Positive regulation of protein-containing complex assembly	GO:0031334	15 (105)	4.97E-02
	LSL	DNA replication	GO:0006260	21 (172)	1.02E-02
		Histone modification	GO:0016570	29 (307)	3.28E-02
3	LB	Purine-containing compound transmembrane transport	GO:0072530	5 (11)	2.16E-03
		Carbohydrate metabolic process	GO:0005975	21 (361)	2.15E-02
	LSL	Negative regulation of autophagy	GO:0010507	10 (44)	1.03E-02
		Carbohydrate derivative transport	GO:1901264	10 (45)	1.28E-02
		Negative regulation of macroautophagy	GO:0016242	7 (21)	1.69E-02
		Carbohydrate metabolic process	GO:0005975	32 (361)	2.79E-02
		Ganglioside metabolic process	GO:0001573	6 (16)	3.44E-02
4	LB	Reactive oxygen species metabolic process	GO:0072593	16 (123)	1.90E-03
		Protein refolding	GO:0042026	4 (6)	2.17E-02
		Protein folding	GO:0006457	13 (102)	2.43E-02
		Hydrogen peroxide metabolic process	GO:0042743	6 (19)	2.71E-02
		Defense response to Gram-negative bacterium	GO:0050829	7 (28)	2.87E-02
		Negative regulation of apoptotic process	GO:0043066	32 (464)	2.89E-02
		DNA replication	GO:0006260	17 (172)	3.84E-02
		Hydrogen peroxide catabolic process	GO:0042744	5 (13)	4.85E-02
	LSL	Inflammatory response	GO:0006954	33 (293)	3.07E-06
		Regulation of cytokine production	GO:0001817	32 (352)	8.57E-04
		Cytokine production	GO:0001816	32 (357)	1.17E-03
		Positive regulation of cytokine production	GO:0001819	23 (217)	2.71E-03
		Leukocyte differentiation	GO:0002521	29 (321)	3.37E-03
		Regulation of cell adhesion	GO:0030155	33 (402)	5.51E-03
		Mononuclear cell differentiation	GO:1903131	24 (243)	5.64E-03
		Regulation of cell-cell adhesion	GO:0022407	22 (214)	7.55E-03
		T cell selection	GO:0045058	8 (31)	1.20E-02
		'de novo' post-translational protein folding	GO:0051084	6 (16)	1.59E-02

### Expression pattern of genes involved in ossification events

**Fig. 6:**
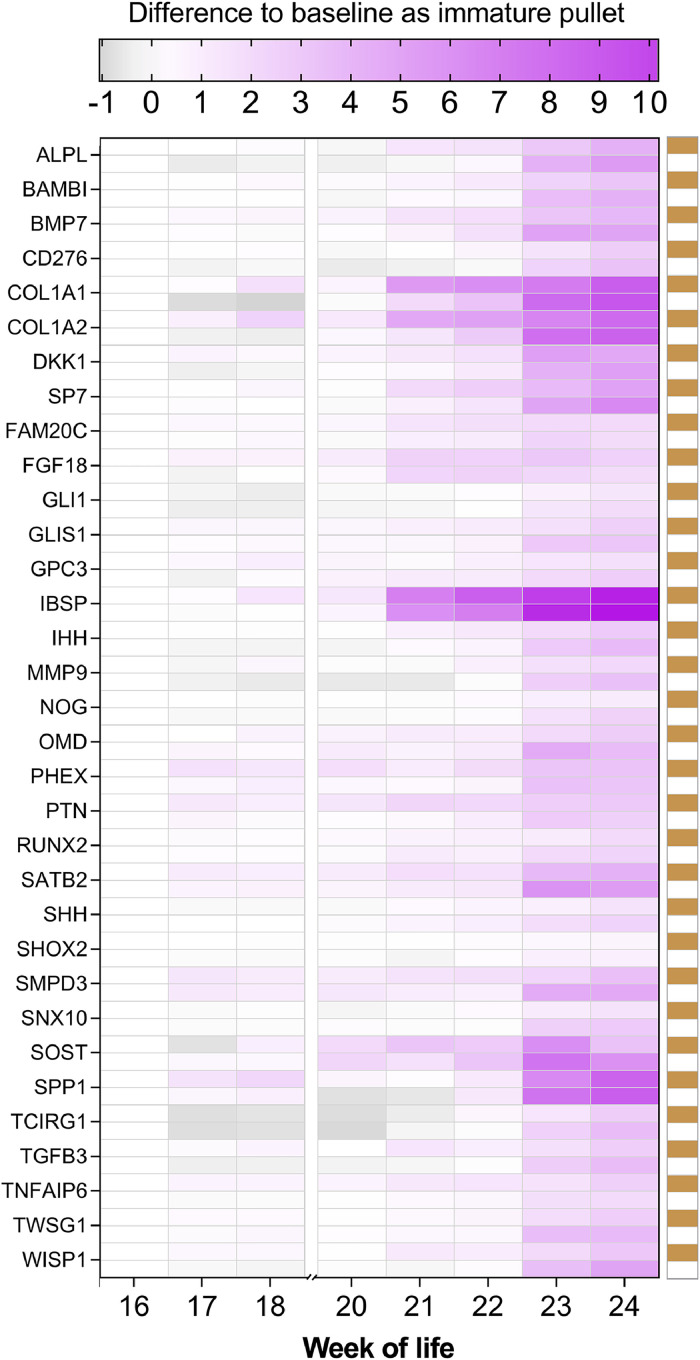
Heatmap displaying temporal expression patterns of genes related to ossification processes in the femur medullary bone of laying hens. Genes assigned to ossification according to STEM profile 1 in both LB (brown squares) and LSL hens (white squares) were used. Relative differences to the immature pullet stage are displayed per laying hen strain throughout the experimental period until week 24. Lowered expression relative to week 16 is shown in grey, increased expression relative to week 16 is shown in purple. Data referring to week 19 were not included.

Fig. 6 shows expression changes of genes throughout the experimental period relative to their baseline level at the immature pullet stage. The statistical differences in gene expression between weeks 16 and 24 per strain are listed in Supplementary Table 5 and Supplementary Table 6. The resulting heatmap emphasizes the temporal dynamics rather than the absolute expression levels. Represented genes affiliated with GO:0001503 are involved in ossification events, i.e., the formation of medullary bone in femur. In general, the expression of these genes increased strongly over time in both laying hen strains. The analysis revealed an induced expression of known key factors, such as ***ALPL*** (Alkaline Phosphatase), ***DKK1*** (Dickkopf WNT Signaling Pathway Inhibitor 1), ***OMD*** (Osteomodulin), ***PHEX*** (Phosphate Regulating Endopeptidase X-Linked), ***SMPD3*** (Sphingomyelin Phosphodiesterase 3), and ***SPP1*** (Osteopontin, Secreted Phosphoprotein 1). Moreover, structural proteins encoded by ***COL1A1*** (Collagen Type I Alpha 1 Chain), ***COL1A2*** (Collagen Type I Alpha 2 Chain), and ***IBSP*** (Integrin Binding Sialoprotein) showed pronounced shifts from weeks 16-20 compared to weeks 21-24. The analyses further revealed ligands such as ***FGF18*** (Fibroblast Growth Factor 18) and ***PTN*** (Pleiotrophin) as well as transcription factors such as ***RUNX2*** (RUNX Family Transcription Factor 2), ***SP7*** (Osterix), and ***STAB2*** (SATB Homeobox 2) to be increasingly expressed in medullary bone along maturation. A negative regulator of bone growth, ***SOST*** (Sclerostin), appeared to be of relevance in both laying hen strains.

## Discussion

The current study focused on plasma traits as well as structural and transcriptional changes in bone specimen of high-yielding laying hens during the transition from the pullet to the mature female bird (week 16 to week 24 of life).

The blood Ca levels were consistent with previous work on LB and LSL hens, where the increased dietary Ca intake resulted in increased Ca plasma levels as well as increased Ca utilization from week 16 to week 24 due to the physiological demand of Ca for eggshell formation (Sommerfeld et al., 2020). Additionally, a time-dependent decrease in blood P levels is in line to our previous study (Sommerfeld et al., 2020). According to the present study, the plasma Ca level specifically did not increase before week 22 (LSL) or week 23 (LB) compared to weeks 16-20 of life, although hens were kept at considerable higher dietary Ca levels from week 16 (pre-layer feed) and week 17 (layer feed). It can be assumed that part of the additional dietary Ca was deposited, as cortical bone Ca contents increased continuously. The mineralization processes in the medullary bone, however, were strongly induced in both LSL and LB strains not before week 23. At this time, 95 % of LSL and 80 % of LB hens have already produced eggs in this study, which challenges the common assumption that the deposition of calcium phosphates (**CaP**) within the medullary bone compartment is already formed 2 weeks before the first egg laying (Nys and Le Roy, 2018). Recent research indicates that the rise in blood estradiol levels in high-performing commercial laying hen strains occurs close to the onset of laying, i.e., the hen’s first egg, which may account for the rather late development of mineral reserves in the medullary bone (Hanlon at al., 2022a). The results of the current study imply that this temporal shift is enabled at the expense of the cortical Ca reservoirs, which preceded the formation of the mineral storage in medullary bone. Although the relative femur ash content increased during the experimental period in this study, this increase may be attributed to the accumulation of carbonate or readily adsorbed Ca ions (Von Euw et al., 2019). The prioritized Ca accretion and the reduced content of P found in cortical femur resulted in a considerable increase of the Ca-P ratio from 2.02 (LSL, LB) in week 16 to 2.16 (LSL) and 2.17 (LB) in week 24. This observation goes along with the high Ca content in the feed and indicates altered metabolic routes for future eggshell formation. The situation in the medullary bone was different as the Ca-P ratio rose from 0.93 (LSL) and 1.13 (LB) in immature pullets (week 16) to a Ca-P ratio of 1.95 (LSL, LB) at the onset of egg laying (week 23), indicating the successful formation of the labile and easily mobilized Ca source. Notably, the used cortical and medullary bone material did not undergo a de-fatting process for ash and mineral quantification (Shastak et al., 2012). It is assumed that the measured P content does not exclusively reflect the inorganic, mineral content of the specimen, but could represent the P fraction from both mineral and soft tissue components. The initial P content of medullary bone material might therefore also refer to the variable abundance of the phospholipid fraction. The achieved Ca-P ratio reflects the rapid transformation of this bone compartment, suggesting the successful deposition of CaP by week 23.

The plasma level of the bone resorption marker beta CTX-1 peaked in week 17-21 (LSL) and week 18-21 (LB) before sharply declining in weeks 22-24 (LSL) and weeks 23-24 (LB). The observed initial increase in beta CTX-1 indicates active bone turn-over, e.g., osteoclast activity, and breakdown of organic matrix. Indeed, the samples taken in this experiment at the immature pullet stage (week 16) did represent bone marrow being highly hematopoietic. The maturity-driven remodelling causes the medullary cavity to be increasingly occupied by medullary bone (Whitehead, 2004). In weeks 22/23, the observed sharp decline of beta CTX-1 levels suggests that the bone matrix degradation was rather low, i.e., that a state of net deposition was achieved. An age-related decline in blood beta CTX-1 levels appeared to continue, as high-yielding LB hens showed lower beta CTX-1 levels in week 49 compared to week 29 (Habig et al., 2021).

Plasma estradiol remained low until week 19 but showed a sharp rise from week 20 (LSL) and week 21 (LB), peaking at week 21 (LSL) and week 23 (LB), respectively. In fact, blood estradiol levels were increased >10-fold in both strains during the transition period of 16 to 24 weeks. The overall blood estradiol profile is in agreement with previous results, where a significant increase was observed between weeks 16 and 24 in laying hens (Omotoso et al., 2021). Furthermore, recurring blood estradiol peaks have been assumed (Hanlon et al., 2022a). Once the hen enters sexual maturity, estradiol action involves the integration and coordination between follicular development in the ovaries, liver metabolism, mineral homeostasis, and bone physiology as reviewed elsewhere (Hanlon et al., 2022a). Elevation in blood estradiol levels during maturation in female chickens was reported to be positively correlated with medullary bone mineral density (**BMD**) and negatively correlated with cortical BMD in tibia (Hanlon et al., 2022b). However, bone ash levels correlated strongly with bone mineral content measurements in laying hens (Robison and Karcher, 2019). The present study showed both increasing blood estradiol and bone ash levels throughout the observation period, indicating a positive relationship between blood estradiol and Ca storage in cortical bone, as also observed in tibia and femur when comparing juvenile and mature hens (Yue et al., 2024).

Previous reports have consistently demonstrated that estradiol exerts a dual regulatory effect on bone metabolism in female chickens (Ohashi et al., 1991; Oursler et al., 1991). In fact, estrogen promotes bone formation and suppresses bone resorption via impacting on osteoblasts (Ernst et al., 1988) and osteoclastic activity (Nakamura et al., 2007; Streicher et al., 2017). Based on the identified STEM results, the profiles 1-4 mirrored similar trends in LB as well as LSL laying hens, suggesting similar gene expression patterns in both strains to facilitate future CaP deposition. The gene expression pattern might follow a diurnal and nocturnal pattern that correlates with hormonal fluctuations and consequently daily laying cycles (Kerschnitzki et al., 2014; Nys and Roy, 2018). However, sampling time of tissues was standardized to minimize confounding factors. According to the performed analyses on GO terms, the STEM profile 1 indicated an overall increase of transcripts associated with bone formation and mineralization in both LB and LSL strains. Indeed, the formation of medullary bone is fundamental in this period of life. Ossification processes are crucial for the ontogenetic development of medullary bone in laying hens. The transcriptomic analysis has identified 33 genes exhibiting increased abundance during the transition from the immature pullet stage to the onset of oviposition in LB and LSL strains. These genes are implicated in both intramembranous and endochondral ossification pathways, ultimately facilitating medullary bone formation to support osteoblast proliferation and subsequent dynamics in mineralization (Parreira et al., 2023). Conversely, genetic polymorphisms within these identified genes may contribute to individual phenotypic variation in medullary bone development, structural characteristics, and its capacity as a Ca reservoir, thus presenting potential targets for selective breeding strategies (Bishop et al., 2000). Single nucleotide polymorphisms in genes critical for bone homeostasis, including ALPL, DKK1, PHEX, and COL1A1, are associated with osteoporosis and related skeletal pathologies in humans. For example, ALPL mutations disrupt bone mineralization in hypophosphatasia, while COL1A1 variants impair collagen integrity in osteogenesis imperfecta (Alonso et al., 2020; Mäkitie et al., 2019). In chicken, strain-specific regulatory mechanisms influence bone metabolism, as evidenced by allele-specific expression of ALPL in LB and LSL laying hens (Iqbal et al., 2023), which correlates with divergent P utilization and skeletal adaptations. Furthermore, the expression of RUNX2, a master regulator of osteogenesis, is dynamically controlled by epigenetic mechanisms during skeletal maturation. DNA methylation at the Runx2 promoter region and miRNA networks (e.g., miR-23a, miR-204) collectively modulate RUNX2 activity, balancing osteoblast differentiation and bone formation in development and disease (Han and Fan, 2021; Kawa et al., 2024).

In addition, results revealed that the expression of a number of candidate genes involved in mineral utilization of bone compartments, such as estradiol receptor 1 (***ESR1***) and estradiol receptor 2 (***ESR2***), did not show a time-dependent progression in the investigated medullary bone. Their expression levels were correspondingly rather low in both strains throughout the experimental period. Moreover, the fibroblast growth factor 23 (***FGF23***) was not expressed in this experimental setting. Other studies, however, reported the presence of FGF23 in both cortical and medullary bone along maturation or egg-laying cycle (Hadley et al., 2016; Yue et al., 2024).

The downstream analysis of STEM profile 2 identified blood vessel remodelling (LB) among the regulated biological processes, where transcript abundances initially increased until week 20, decreased until week 22, and finally plateaued. This might be related to remodelling mechanisms to ensure efficient mineral deposition in the medullary bone. The inclusion of *BCR* and *EPAS1* in this biological process reflects aspects of vascular remodelling as well as angiogenesis taking place in the medullary bone at the onset of egg laying. In particular, *EPAS1* drives angiogenesis through hypoxia-responsive pathways and acts as a transcription factor to regulate a variety of genes for vascular formation (Takeda et al., 2004), while e.g. *MDM2* and *RBPJ*-associated mechanisms contribute to structural and functional vascular remodelling. Interestingly, blood flow rates in the nutrient-supplying arteries of femora were higher in 4-8 month old laying hens than in non-laying hens and roosters, suggesting extra blood flow required for higher Ca mobilization and deposition in medullary bone during eggshell production (Hu et al., 2021). The active enrichment of histone modification-associated genes in both laying hen strains points to epigenetic regulation as a key mechanism facilitating the dynamic remodelling of the medullary bone. Central to this process is *HDAC4*, which modulates osteoblast differentiation through directly acting on histone acetylation of the promotor region of *RUNX2* and interacting with *MEF2* and *SMAD3* to alter *RANKL* expression (Obri et al., 2014; Yi et al., 2019). The STEM profile 3, whose associated transcripts showed a significant decrease in abundance until week 20 and an increase from week 21, showed a link to carbohydrate metabolic process in both strains, suggesting a role in energy metabolism and transport processes required at onset of laying activity. The dynamic bone remodelling inherently involves elevated cellular energy expenditure for ion transport, matrix synthesis, and resorptive processes. The synchronization of high metabolic flux and active cellular processes required for eggshell formation underscores the increased energy requirements of medullary bone cells. As STEM profile 2, the profile 3 also contains biological processes critical for medullary bone remodelling, including the negative regulation of autophagy, which is notably enriched in LSL laying hens. This regulatory mechanism could facilitate morphological restructuring by supporting the survival and function of both osteoclasts and osteoblasts, thus maintaining the balance between bone formation and resorption (Nollet et al., 2014). The STEM profile 4 described an overall decrease in medullary bone gene expression associated with immune features (LSL) from the immature pullet stage to the onset of lay. The results reflect the tissue’s active role in immune development and haematopoiesis before sexual maturity. Accordingly, the STEM profile 4 comprises immunity-related hub genes, including *CEBPB, IRF4, IRF8*, and *MYD88*, which collectively orchestrate immune homeostasis. *CEBPB*, which was assigned to the STEM profile 4 in both LB and LSL hens, regulates numerous genes such as pro-inflammatory cytokines and antimicrobial peptides involved in both innate and adaptive immunity (Ren et al., 2023). *IRF4* and *IRF8* balance adaptive and innate immunity, where the downregulation of the latter might be involved in RANKL-mediated osteoclastogenesis to ensure the mineral turn-over for eggshell formation (Hrdličková et al., 2009; Zhao et al., 2009). *MYD88* integrates stress signals to modulate inflammation and was also suggested to play an important role in bone resorption (Leite et al., 2015). As hens mature, medullary bone became specialized for Ca storage, and expression signature in early bone marrow referring to immune-related genes decreased accordingly. This is in line with previous observations comparing pullets and mature female chickens, where cell counts of various T cell types decreased after week 15 (Schmucker et al., 2021). However, the numbers of monocytes and heterophils in blood increased throughout the experimental period (Schmucker et al., 2021). Additionally, genes involved in hydrogen peroxide metabolism were part of the STEM profile 4 and were downregulated during maturation in LSL laying hens. Hydrogen peroxide levels in osteoclasts can increase their activity and subsequently enhance bone resorption in a rodent model (Lean et al., 2005). In this study, the observed downregulation of genes like *GPX1*, which breaks down intracellular hydrogen peroxide molecules using glutathione (**GSH**), could facilitate this process by promoting osteoclast-mediated bone turn-over, thus enabling the formation of new medullary bone deposition.

Taken together, the remodelling of the femoral compartments with corresponding successful mineralization of the medullary bone was mapped in laying hens using bone and blood parameters as well as transcriptome analyses. The overall profile of blood Ca, P, beta CTX-1, and estradiol suggest a slightly earlier maturation in LSL compared to LB laying hens, which indicates strain-specific shifts in the temporal reallocation of minerals. Gene expression patterns of medullary bone specimen reflected the fundamental structural turn-over once the female chickens enter sexual maturation. The underlying molecular processes leading to efficient medullary bone formation involve longitudinal increased expression of genes related to ossification and bone development. Comparative analysis of genes expressed in both LB and LSL laying hen strains identified 33 genes that likely serve as pivotal regulators of the functional development of the maturing medullary bone. These factors can be further investigated to enhance molecular breeding, nutritional management, and early intervention strategies. This ultimately enables rapid phenotyping in larger flocks to optimize mineral supply in sensitive developmental stages.

SUPPLEMENTAL MATERIAL

The xlsx-files contain information to feed ingredients (Table S1), DEG between strains per time point (Table S2), DEG across adjacent time points per strain (Table S3, Table S4), gene symbols related to STEM profiles 1-4 per strain (Table S5, Table S6), and term names including affiliated gene symbols according to GO annotations based on STEM profiles (Table S7).

## CRediT authorship contribution statement

**Michael Oster:** Writing – review & editing, Writing – original draft, Visualization, Methodology, Investigation, Funding acquisition, Formal analysis, Data curation, Conceptualization. **Hiba Qasir:** Writing – review & editing, Validation, Investigation, Formal analysis, Data curation. **Henry Reyer:** Writing – review & editing, Writing – original draft, Validation, Methodology, Investigation, Formal analysis, Data curation. **Siriluck Ponsuksili:** Writing – review & editing, Supervision, Resources, Funding acquisition, Conceptualization. **Nares Trakooljul:** Writing – review & editing, Methodology, Investigation, Data curation. **Vera Sommerfeld:** Writing – review & editing, Methodology, Investigation, Data curation, Conceptualization. **Markus Rodehutscord:** Writing – review & editing, Resources, Funding acquisition, Conceptualization. **Klaus Wimmers:** Funding acquisition, Project administration, Resources, Supervision, Writing – review & editing.

## Disclosures

The authors declare the following financial interests/personal relationships which may be considered as potential competing interests:

Klaus Wimmers reports financial support was provided by Deutsche Forschungsgemeinschaft (DFG, German Research Foundation). If there are other authors, they declare that they have no known competing financial interests or personal relationships that could have appeared to influence the work reported in this paper.

## Appendix Supplementary materials

Image, application 1

Image, application 2

Image, application 3

Image, application 4

Image, application 5

Image, application 6

Image, application 7

## Footnotes

Supplementary material associated with this article can be found, in the online version, at doi:10.1016/j.psj.2025.105798.
